# uPAR enhances malignant potential of triple-negative breast cancer by directly interacting with uPA and IGF1R

**DOI:** 10.1186/s12885-016-2663-9

**Published:** 2016-08-08

**Authors:** Michaela C. Huber, Rebecca Mall, Herbert Braselmann, Annette Feuchtinger, Sara Molatore, Katrin Lindner, Axel Walch, Eva Gross, Manfred Schmitt, Natalie Falkenberg, Michaela Aubele

**Affiliations:** 1 Institute of Pathology, Helmholtz Zentrum München, German Research Center for Environmental Health, Ingolstaedter Landstrasse 1, 85764 Neuherberg, Germany; 2 Research Unit of Radiation Cytogenetics, Helmholtz Zentrum München, German Research Center for Environmental Health, Ingolstaedter Landstrasse 1, 85764 Neuherberg, Germany; 3 Research Unit of Analytical Pathology, Helmholtz Zentrum München, German Research Center for Environmental Health, Ingolstaedter Landstrasse 1, 85764 Neuherberg, Germany; 4 Tumor Genetics Unit, Department of Obstetrics and Gynecology, Technische Universität München, Ismaninger Strasse 22, 81675 München, Germany; 5 Clinical Research Unit, Department of Obstetrics and Gynecology, Technische Universität München, Ismaninger Strasse 22, 81675 München, Germany

**Keywords:** IGF-1R, TNBC, Prognostic impact, uPAR interactome, uPAS, uPA system

## Abstract

**Background:**

Due to lack of a targeted therapy for the triple-negative breast cancer (TNBC) patients, it is important to explore this aggressive breast cancer type in more detail and to establish novel therapeutic approaches. TNBC is defined negative for the protein expression of oestrogen receptor (ER), progesterone receptor (PR) and human epidermal growth factor receptor 2 (HER2). One prominent feature of this cancer type is the frequent overexpression of major components of the urokinase-type plasminogen activator system (uPAS) including uPA, its receptor uPAR and the inhibitor PAI-1, which may be valuable as therapeutic targets.

**Methods:**

Direct interactions of uPAR with interactors were demonstrated by immunoprecipitations and proximity ligation assays. For stable knockdowns of target proteins, lentiviral vectors were used and the effects were analysed by immunoblottings and using in vitro cell viability, migration and invasion assays. Immunohistochemical and statistical analyses of biomarkers and clinical parameters were conducted in a TNBC cohort (*n* = 174).

**Results:**

Direct tumour-promoting interactions of uPAR with uPA and the insulin-like growth factor receptor 1 (IGF1R) were shown in TNBC cells and these interactions were significantly reduced (*p* = 0.001) when uPAR was downregulated. The combined knockdown of uPAR and uPA or IGF1R additively and significantly reduced cell viability, migration and invasion of the model cell lines. In TNBC tissue, the complexes formed by uPAR with uPA or with IGF1R significantly correlated with the histological grade (*p* = 0.0019) as well as with cathepsin B and D (*p* ≤ 0.0001) that are implicated in cell invasion and metastasis.

**Conclusions:**

Our outcomes show that not only overexpressed biomarkers promote tumourigenesis, but rather their interactions further potentiate tumour progression. This study emphasises the potential of combined approaches targeting uPAR and its interactors with regard to an improved therapy of TNBC.

**Electronic supplementary material:**

The online version of this article (doi:10.1186/s12885-016-2663-9) contains supplementary material, which is available to authorized users.

## Background

In TNBC, there is a lack of protein expression of the oestrogen receptor (ER) and the progesterone receptor (PR) as well as a weak or absent protein expression of the human epidermal growth factor receptor 2 (HER2) [1]. TNBC is the most aggressive tumour type among breast cancers that is associated with a poor prognosis and occurs in approximately 10 to 20 % of invasive breast cancers [2]. Due to the lack of targeted therapies, the patients are treated systemically leading to severe side effects and besides that the therapy efficacy is limited; therefore novel therapeutic targets are strongly needed. Several research groups revealed insulin receptor (IR), insulin-like growth factor receptor 1 (IGF1R), epidermal growth factor receptor (EGFR), hepatocyte growth factor receptor (c-Met) and in particular the urokinase-type plasminogen activator (uPA) with its receptor (uPAR) to be overexpressed in many tumour entities including TNBC [3–9]. Except uPAR, these transmembrane receptors are activated by the binding of growth factors to their extracellular domain, followed by the formation of homo- and/or heterodimers, which induce phosphorylation of the intracellular receptor domains and recruit further signalling molecules to initiate signalling cascades within the cells [10, 11].

The receptors IR, IGF1R, EGFR and c-Met promote cell proliferation, invasion, survival and metastasis by activating the phosphatidylinositol 3-kinase (PI3K), Akt, mTOR pathway as well as the Ras, Raf, mitogen-activated protein kinase (MAPK), extracellular signal-regulated kinase (ERK) pathway and the signal transducer and activator of transcription (STAT) 3-mediated signalling [4, 12, 13]. uPAR is strongly involved in wound healing, clot lysis, tissue remodeling through binding to and activating pro-uPA, which in turn stimulates further invasion-promoting factors such as plasminogen and pro-matrixmetalloproteases (pro-MMPs) followed by the degradation of the extracellular matrix (ECM) leading to migration and invasion of tumour cells [14]. It has been shown that strongly invasive TNBC cells and respective cell lines use this natural process and enhance their invasive capacity through overexpression of uPAR, uPA or MMPs [9, 15]. Depending on their cellular context and expression levels, IR, IGF1R, EGFR, c-Met and uPAR promote malignancy also through cooperating with each other and may be promising candidates for an improved cancer therapy [13]. Since uPAR is solely associated to the plasma membrane by a glycosylphosphatidyl inositol (GPI) anchor, interactions with membrane-spanning receptors may enable uPAR-mediated intracellular signalling as well.

Considering IGF1R and IR inhibition, many attempts have been made with respect to targeted therapies, including several receptor tyrosine kinase inhibitors (RTKIs), monoclonal humanised antibodies or IGF-binding proteins [3]. However, the therapeutic approach turned out to be less successful than expected. Due to high homology on DNA and protein level [16], a cross talk between IGF1R and IR signalling is supposed to diminish the inhibitory effects [10]. Another challenge is the role of IR, which is significantly involved in glucose metabolism and therefore could not be completely blocked [10]. EGFR-targeted therapeutic antibodies such as cetuximab or panitumumab or the TKIs gefitinib and erlotinib were applied in vitro and in clinical trials with TNBC patients indicating a successful therapy, however, mostly combinational approaches, also with chemotherapeutics, were more effective [17–22]. Anti-c-Met-based therapy using antibodies or tyrosine kinase inhibitors are currently tested in phase II clinical trials in TNBC patients [23–25]. Nevertheless, the success of RTKIs for cancer therapy is also limited due to intrinsic or acquired resistance by cancer cells [26].

In addition, therapeutic approaches have been made towards the uPA system (uPAS) components such as uPA, uPAR or PA inhibitor-1 (PAI-1). In particular in breast cancer, uPA and PAI-1 have been validated as predictive or prognostic biomarkers and in vivo experiments emphasise anti-tumour effects when uPA-uPAR interactions or uPA alone were inhibited [27–30]. uPAR was also knocked down in combination with the RNAi of uPA, HER2 or MMP9 or combined with Trastuzumab in different breast cancer cell lines and in in vivo studies resulting in reduced cell migration, invasion, angiogenesis or proliferation [31–33]. Co-overexpression of uPA, uPAR and IGF1R elevated the malignancy of pancreatic, hepatocellular, rhabdomyosarcoma, colon and breast cancer cells [6, 34–36].

To date, little is known regarding the role of uPAR and IGF1R in TNBC and whether these receptors directly interact with each other. In this study, to investigate in more detail the impact of these receptors in TNBC, uPAR, uPA and IGF1R were stably and simultaneously knocked down in two TNBC cell lines and the effects on in vitro cell migration, invasion, proliferation and on signalling molecules were examined. Furthermore, immunohistochemical analyses and PLA also using TNBC tissue samples (*n* = 174) demonstrate a direct interaction of uPAR with uPA and with IGF1R emphasizing additive effects of those interactions on TNBC tumour progression.

## Methods

### Cell culture

The following human breast cancer cells lines MDA-MB-361 (HTB-27), SKBR3 (HTB-30), T47D (HTB-133) were acquired from American Type Culture Collection (ATCC) and MCF7 (ACC115) cells from German Collection of Microorganisms and Cell Cultures, DSMZ). The BT549 and MDA-MB-231 cell lines are a kind gift from Prof. M. Schmitt, Clinical Research Unit, Department of Obstetrics and Gynecology, Technische Universität München). The BT549, T47D and MCF7 cells were maintained in RPMI 1640 with GlutaMAX (Rosewell Park Memorial Institute medium) supplemented with bovine insulin (10 μg/μl, Sigma, St. Lois, MO, USA). The SKBR3 and MDA-MB-361 cells were maintained in DMEM with GlutaMAX (Dulbecco Modified Eagles medium) that was additionally supplemented with non-essential amino acids (Life Technologies, Darmstadt, DE) for cultivation of MDA-MB-231 cells. Both media were supplemented with 10 % fetal calf serum (FCS, Invitrogen, Carlsbad, CA, USA) and 0.25 % of each penicillin and streptomycin (Life Technologies, Darmstadt, DE). The cells were maintained in a water humidified 37 °C incubator with 5 % CO_2_. The last cell line authentication was conducted before starting the experiments as described previously [37].

### Lentiviral transductions of breast cancer cell lines

SMARTchoice™ lentiviral shRNA vectors (GE Healthcare Lafayette, CO, USA) were used for transductions for RNAi of uPAR (VSH6063, SH-006388-01, −02, −03), uPA (VSH6063, SH-006000-01, −02, −03), IGF1R (VSH6063, SH07-003012-04, −05, −06). For efficient knockdown of the target proteins, a pool of three viral particles (targeting three different fragments within the RNA sequence of the target protein), each at a multiplicity of infection (MOI) of 30, was used. All viral particles were tested for knockdown specificity and efficiency before starting the RNAi experiments. A total of 3.0 × 10^4^ BT549 or MDA-MB-231 cells were seeded into each well of a 12-well plate and after 42 h infected with the lentiviral vectors for the knockdown of uPAR (uPAR_RNAi), of uPA (uPA_RNAi) and of IGF1R (IGF1R_RNAi). For a successful knockdown of the strongly overexpressed uPAR, respective infection was repeated several times as described [38]. A vector containing a non-targeting sequence (SMARTvector 2.0 non-targeting particle) was used as negative control and a vector for GAPDH knockdown was used as positive control (SMARTvector 2.0 Human GAPD). For enhancing the infection, 2 μg/ml polybrene (Invitrogen, Carlsbad, CA, USA) was added to each approach as described [39]. All infections were conducted in triplicates.

### Quantitative reverse transcription-PCR (qRT-PCR)

The RNA isolation, TaqMan assays and analysis were conducted as described [40]. Quantitative PCR was conducted in triplicates using TaqMan probes: uPAR (Hs00958880_m1), uPA (Hs01547054_m1) and IGF1R (Hs00609566_m1, Thermo Fisher, Waltham, Massachusetts, USA). The mRNA expression levels were calculated by the 2^–ΔΔCT^ method and normalised to the control (HPRT1) and to MCF7 cells (calibrator).

### Western blot analysis

The analysis and quantification of protein expressions or phosphorylations of uPAR [41], uPA, PAI-1, IGF1R, IR, c-Met, Paxillin, 44/42 MAPK, HER2, PR, ER, STAT3, p27^Kip1^, MMP2, MMP 9, GAPDH and Tubulin were analysed using primary antibodies (Additional file 1: Table S1) as described previously [42].

### Immunoprecipitations

To determine potential interaction partners of uPAR, diverse immunoprecipitation protocols were established using the goat polyclonal anti-uPAR antibody (AF807, R&D Systems, Minneapolis, MN, USA) for 60 min at 4 °C based on previously described procedure [43]. As negative control, a polyclonal goat isotype antibody was applied. The precipitated proteins were eluted with Laemmli buffer (2×) and further used for Western Blot analysis.

### Wound scratch migration assay

To analyse the knockdown effects on in vitro cell migration, migration assays were conducted in six-well plates under serum-reduced conditions (0,1 % FBS) and the open areas were quantified using TScratch software as described [39]. The migration assays were conducted in triplicates and the Student’s *t*-test was used for statistical analysis.

### Invasion assays

To analyse the knockdown effects on in vitro cell invasion, 2.0 × 10^4^ control or infected BT549 or MDA-MB-231 cells were seeded in serum-reduced medium (0,1%FBS) in matrigel-coated chambers, inserted into 24-well cell culture plates and incubated for 48 h at 37 °C. The invasion assays were conducted according to manufacturers’ protocol and quantified by counting the invaded cells in 13 representative images. The invasion assays were conducted in triplicates and the means of each approach were quantified in relation to the mock control. The Student’s *t*-test was used for statistical analysis.

### WST-1 cell proliferation assay

To analyse the knockdown effects on in vitro cell proliferation, 1.0 × 10^4^ control or infected BT549 or MDA-MB-231 cells per well were seeded in a 96-well culture plate and the water soluble tetrazolium WST-1 assay (Roche Diagnostics, Mannheim, DE) was conducted in quintuplicate as described previously [37]. The Student’s *t*-test was used for statistical analysis.

### Generation of formalin-fixed and paraffin-embedded (FFPE) cell line blocks

To optimise the analysis of protein expressions and of protein complexes in cell lines and tumour samples, control and uPAR-depleted MDA-MB-231cell line blocks were generated as described previously [44].

### Patients and tumour specimens

Formalin-fixed, paraffin-embedded breast cancer tissues of the TNBC type (*n* = 174) were collected at the Department of Gynecology and Obstetrics, Klinikum rechts der Isar, Technische Universität München, Germany. Written informed consent for the use of tissue samples for research purposes was obtained from all the patients. Approval for use of the tumour samples was given from the Ethics Committee of the Medical Faculty of the Technische Universität München, Germany.

The negative or only low protein expression of HER2 and of the steroid hormone receptors (ER, PR) in tumour tissues was verified by immunohistochemical analysis as described below. The other parameters of the 174 tumours are as following, in total, 90 tumours were node-negative. Concerning tumour size, 52 tumours were less than 2 cm, 93 were between 2 and 5 cm, and 29 tumours were more than 5 cm in size. The most tumours were classified as grade 3 (*n* = 150), followed by 21 cases as grade 2, and 3 cases as grade 1 [45]. From 68 patients, data concerning breast cancer 1 mutation were available. The median follow-up of patients was 57 months (max. 244 months), with 52 (30 %) patients suffering from metastases within the period of clinical follow-up. All tumour patients were surgically treated and 108 of the patients received adjuvant chemotherapy.

### Generation of tissue microarrays (TMAs)

TMAs were generated with a tissue-arraying instrument (Beecher Instruments Inc., Silver Spring, MD, USA) as described [46]. Three micrometer thick sections were cut from the TMA blocks and both, the TMA and the punched block were re-examined to validate representative sampling.

### Immunohistochemical (IHC) analysis

Immunohistochemical analysis was performed on 3 μm thick sections of the FFPE cell blocks and of tumour tissues using an automated stainer (Discovery XT) and a DAB Map kit (both Ventana Medical Systems, Tucson, AZ, USA) as described [46]. The applied antibodies targeting uPAR, uPA, PAI-1, IGF1R, IR, c-Met, HER2, PR, ER, Plasminogen, Ki67, uPARAP, PTEN, p27^Kip1^, Cathepsin B and D are listed in Additional file 1: Table S1. The stained TMA specimens were assessed and scored by two independent observers using a 4-point scale (0–3+) [46].

### Proximity ligation assay (PLA) technique on FFPE sections

For the visualisation of protein complexes with interaction partners of uPAR, the PLA technique was conducted on cell line blocks and on TMAs from tumour samples using the DUOLink^TM^ kit (OLINK, Uppsala, S) according to the manufacturer’s instruction as described [39, 47]. The primary antibodies targeting uPAR, uPA and IGF1R were the same, which were applied for IHC analysis. For the quantification of PLA, the slides were scanned and signals were evaluated as described previously [47]. For each protein complex, signals of three visual fields per figure were calculated and subjected to statistical analysis.

### Statistical analysis of tumour tissues

The correlations between experimental parameters and histopathological parameters were examined with Spearman’s rank correlation test. In all tests, statistical significance was considered when *p* ≤ 0.05.

## Results

### uPAR, IGF1R and c-Met are significantly co-overexpressed in TNBC samples and breast cancer cell lines

**Fig. 1:**
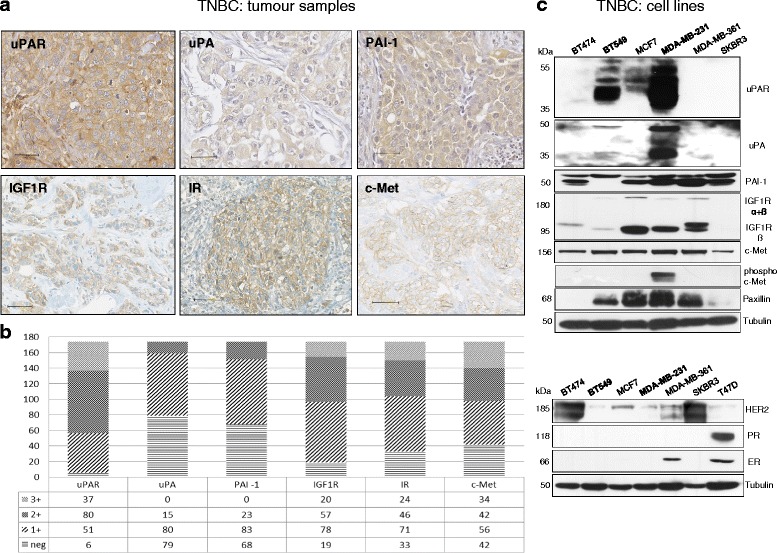
Co-overexpression of uPAS components and tumour-promoting proteins in TNBC samples and cell lines. **a** Immunohistochemical analysis of tumour samples showing positive expression and localisation of the proteins of interest: uPAR, uPA, PAI-1, IGF1R, IR and c-Met, bar: 50 μm and **b** Protein expressions of uPAR, uPA, PAI-1, IGF1R, IR and c-Met in the TNBC cohort (*n* = 174). **c** Immunoblottings of uPAR, uPA (supernatant), PAI-1, IGF1R, (phospho) c-Met, HER2, ER, PR in two TNBC cell lines: BT549 and MDA-MB-231 and in the breast cancer cell lines: BT474, MCF7, MDA-MB-361, SKBR3 and T47D. Tubulin was used as loading control

To select TNBC tumour samples for this study, the expression of markers of the uPA system and related tumour-promoting proteins was determined by IHC analysis. The three major components of the uPAS including uPA, uPAR and PAI-1 as well as IGF1R, IR and c-Met are highly expressed and mostly localised on the cell membrane and in the cytoplasm (Fig. 1a and b). In contrast, ER, PR and HER2 are not or moderately expressed in this TNBC cohort. In addition, for the identification of model breast cancer cell lines representative for TNBC, the protein expressions were determined by immunoblotting. Out of seven breast cancer cell lines analysed here, only the BT549 and MDA-MB-231 cells (strongly) express uPA and uPAR. The IGF1R and the paxillin protein expression levels are higher in MDA-MB-231 than in BT549 cells and the phosphorylated c-Met could only be detected in MDA-MB-231 cells, whereas the protein expressions of ER and PR were absent and of HER2 very weak in these TNBC cell lines (Fig. 1c).

### The combined knockdown of uPAS components and of IGF1R significantly impairs tumour cell proliferation, migration and invasion in vitro

To investigate whether the knockdown of uPAS components and associated signalling proteins impairs the tumour progression of BT549 and MDA-MB-231 cells in vitro, uPAR, uPA and IGF1R were transiently downregulated, also in combination, in BT549 cells using several targeting and control small interfering RNAs (siRNAs, Additional file 2: Figure S1a and Additional file 3: Figure S2a). Transient RNAi of the target proteins and/or in combination significantly reduced in vitro cell viability, migration and invasion 48 h post-transfection (Additional file 2: Figure S1b-e and Additional file 3: Figure S2b-e). The expression of the invasion-promoting proteins MMP2, MMP9 or paxillin and the phosphorylation of STAT3 were reduced following uPAR RNAi alone or in combination with RNAi of uPA or IGF1R (Additional file 2: Figure S1b and Additional file 3: Figure S2b).

**Fig. 2:**
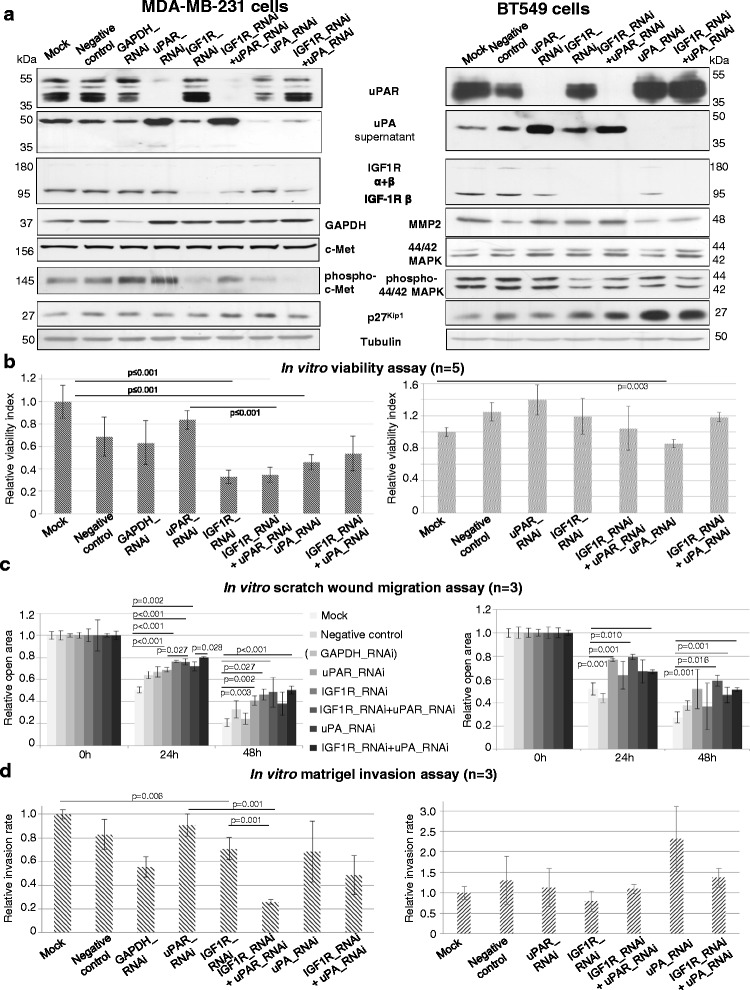
Co-RNAi of uPAS components and of IGF1R significantly reduces malignancy of BT549 and MDA-MB-231 cells. **a** Representative immunoblottings of uPAR, uPA (supernatant), IGF1R, GAPDH, (phospho) c-Met, MMP2, (phospho) 44/42 MAPK and p27^Kip1^ are shown. Tubulin was used as loading control. **b** WST-1 assay was conducted 48 h (*n* = 5), **c** Scratch wound assays 24 h and 48 h (*n* = 3), **d** Matrigel invasion assays were conducted 48 h after starting the experiment (*n* = 3). The quantifications were determined relative to mock

For long-term analysis of tumour progression following the knockdown of uPAS components and IGF1R with regard to an improved breast cancer therapy, the target proteins were stably downregulated. The cells were incubated for up to 8 weeks and the effects were determined using in vitro assays and immunoblottings. Successful and specific knockdown of the target proteins uPAR, IGF1R and uPA was confirmed by qRT-PCR (Additional file 3: Figure S2f) and by Western blots (Fig. 2a). GAPDH-RNAi was used as a positive control of optimal infection conditions for the cell lines and a non-targeting shRNA sequence within the lentiviral vector was used as negative control. GAPDH-RNAi led to a strong inhibition of proliferation of BT549 cells; therefore, these approaches could not be used for respective assays and are not shown. Apart from that, the used control shRNAs do not show any unspecific effects regarding the protein and RNA levels in both cell lines (Fig. 2a and Additional file 3: Figure S2f).

In MDA-MB-231 cells, RNAi of IGF1R or in combination with uPAR RNAi or uPA RNAi led to a strongly reduced phosphorylation of c-Met (Fig. 2a, left). In BT549 cells, the RNAi of IGF1R or combined with RNAi of uPAR or uPA led to a reduced phosphorylation of 44/42 MAPK (Fig. 2a, right). Following uPA RNAi or co-RNAi of uPA and IGF1R in BT549 cells, the MMP2 protein expression was diminished (Fig. 2a, right). In both cell lines, the cell cycle inhibitor p27^Kip1^ was induced following single IGF1R knockdown or combined with RNAi of uPA or uPAR (Fig. 2a), whereas no alteration on phosphorylation of STAT3 or Akt was observed (data not shown).

In comparison to the mock control, the cell proliferation was significantly (*p* ≤ 0.001) reduced in MDA-MB-231 cells following RNAi of IGF1R (−70 %) or uPA (−55 %) and following co-RNAi of uPAR and IGF1R (−65 %) or of uPA and IGF1R (−50 %) (Fig. 2b, left). In BT549 cells, compared to the mock control, only uPA-depleted cells showed a significantly reduced in vitro cell proliferation (−16 %, *p* = 0.003, Fig. 2b, right).

Furthermore, compared to MDA-MB-231 mock cells, the in vitro migration was significantly reduced following the RNAi of uPAR (−18 %, *p* < 0.001) and/or IGF1R (−25 %, *p* < 0.001, respectively) and uPA (−20 %, *p* = 0.002) (Fig. 2c, left, 24 h after starting the assay). More importantly, following co-RNAi of IGF1R and uPAR and compared to uPAR RNAi alone, the cell migration was additively and significantly reduced (−8 %, *p* = 0.027) as well as following co-RNAi of uPA and IGF1R compared to uPA RNAi alone (−8 %, *p* = 0.028) (Fig. 2c, left). Considering the effects 48 h after starting the in vitro migration assay and compared to the mock control, the RNAi of uPAR (−20 %, *p* = 0.003), of IGF1R (−25 %, *p* = 0.002) and of both receptors (−28 %, *p* = 0.027) or of IGF1R with uPA (−30 %, *p* < 0.001) significantly diminished the in vitro migration (Fig. 2c, right). Compared to BT549 mock control cells, the in vitro migration was significantly reduced following uPAR RNAi (−20 %, p = 0.001) or co-RNAi of uPA and IGF1R (−22 %, *p* = 0.001) as well as following co-RNAi of uPA and IGF1R (−22 %, *p* = 0.010) (Fig. 2c, right, 24 h after starting the assay). Compared to IGF1R RNAi alone, after the co-RNAi of uPAR and IGF1R (−16 %) the in vitro BT549 cell migration was additively reduced but not statistically significant (Fig. 2c, right, 24 h after starting the assay). Regarding the effects 48 h after starting the in vitro migration assay and compared to the mock control, the RNAi of uPA has a significant and reducing effect on in vitro BT549 cell migration (−15 %, *p* = 0.015), as well as the co-RNAi of IGF1R with uPAR or uPA (−28 % or −20 %, *p* = 0.001, respectively; Fig. 2c, right).

Concerning in vitro cell invasion, compared to MDA-MB-231 mock control cells, the RNAi of IGF1R significantly impaired cell invasion (−30 %, *p* = 0.006, Fig. 2d, left). Compared to RNAi of uPAR or IGF1R alone, the co-RNAi of uPAR and IGF1R significantly and additively reduced cell invasion (−65 % or −45 % respectively, *p* = 0.001) and showed the strongest effect of the analysed approaches (Fig. 2d, left). In BT549 cells, the in vitro cell invasion was not changed following RNAi of the target proteins or was elevated due to uPA RNAi (Fig. 2d, right).

In summary, the long-term RNAi of IGF1R and the combined RNAi with uPAR led to the most efficient reduction of in vitro proliferation, migration and invasion in both TNBC cell lines.

### uPAR directly interacts with uPA and with IGF1R in vitro

**Fig. 3:**
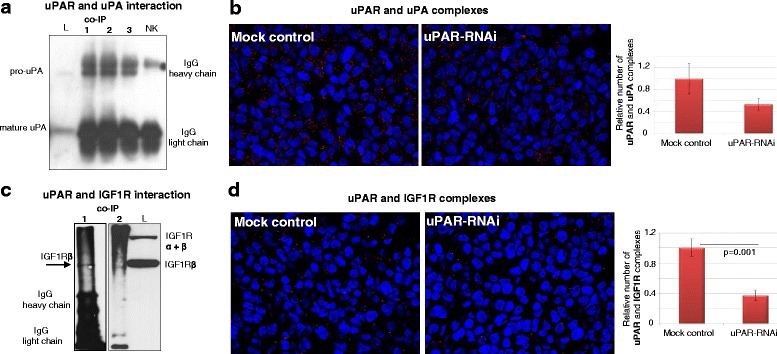
uPAR directly interacts with uPA and IGF1R. **a** Western blot analyses of co-immunoprecipitation replicates (co-IP 1–3) of uPAR with uPA or with IGF1R (**c**), the lysate (L) of MDA-MB-231 cells was used as positive control and a non-targeting isotype antibody was used as negative control (NK) **b** PLA and quantification of protein-protein complexes of uPAR with uPA or with IGF1R (**d**) on cell block sections from mock and uPAR-depleted cells (uPAR-RNAi)

Following the optimisation of immunoprecipitation protocols for uPAR and interaction partners in MDA-MB-231 cells, uPA, which is already known as a direct interactor of uPAR, was successfully precipitated (Fig. 3a). Based on this, novel direct uPAR interaction partners could be detected in MDA-MB-231 cell lysates. IGF1R has also been shown to be associated with uPAR and to promote cellular invasion and migration in cancer cells of the pancreas and colon as well as of rhabdomyosarcoma cells in vitro [34–36]. Here, using further optimised protocols for immunoprecipitations, the direct interaction of uPAR with IGF1R could be demonstrated (Fig. 3c). Furthermore, a relatively new technology for the visualisation of interactions based on formation of protein-protein complexes, the proximity ligation assay (PLA), was conducted on FFPE sections derived from mock and uPAR-depleted MDA-MB-231 cells (Additional file 4: Figure S3a). Using PLA, the complexes of uPAR with uPA could be shown and their number was reduced following uPAR RNAi (Fig. 3b). Moreover, the complex formation of uPAR with IGF1R was successfully demonstrated and their number was significantly decreased when uPAR was downregulated (−62 %, *p* = 0.001, Fig. 3d). Weakly uPAR-positive and strongly IGF1R-positive MCF7 cells (Fig. 1c and Additional file 4: Figure S3b) or SKBR3 cells, which are almost negative for the protein expression of both receptors (Fig. 1c and Additional file 4: Figure S3d) were used as controls for visualisation of uPAR-IGF1R complexes by PLA. In comparison with MDA-MB-231 mock cells, a significantly reduced number of complexes was detected in MCF7 cells (−60 %, *p* = 0.001, Additional file 4: Figure S3c) that was further reduced in SKBR3 cells (−85 %, Additional file 4: Figure S3e).

### uPAR directly interacts with uPA and with IGF1R in tumours and promotes the malignancy of TNBC

**Fig. 4:**
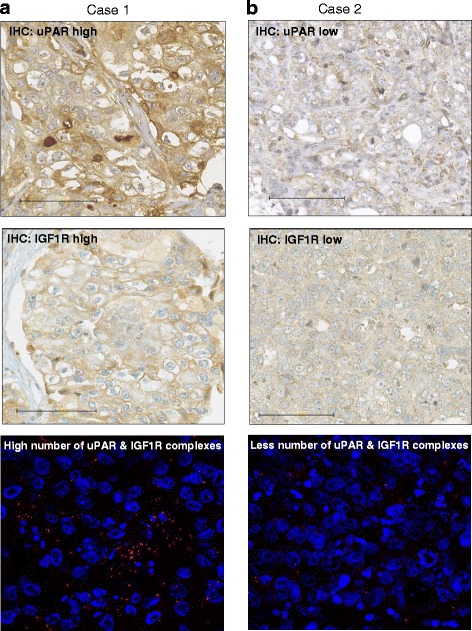
The uPAR and IGF1R complexes correlate with their co-overexpression in TNBC samples. **a** Immunohistochemical analyses and visualisation of uPAR and IGF1R in the same tumour sample expressing the target proteins at a high (score 3+) or (**b**) at a low (score 1+) level, bar: 100 μm

In the TNBC cohort used for this study, several uPAS components and tumour-relevant markers have been revealed to be significantly co-overexpressed (Table 1). In particular, uPAR significantly correlated with IGF1R (*p* = 0.0011), the cathepsins B (*p* = 0.0034) and D (*p* = 0.0075), c-Met (*p* = 0.0229), IR (0.0215) and plasminogen (*p* = 0.0105). uPA significantly correlated with PAI-1 (*p* ≤ 0.00001), cathepsin D (*p* = 0.0187), plasminogen (*p* = 0.0237) and inversely with Ki67 (*p* = 0.0004) (Table 1). IGF1R significantly correlated with IR (*p* = 0.0020), cathepsin B (*p* = 0.0002), plasminogen (*p* = 0.0237) and several other biomarkers (Table 1). No association between breast cancer 1 mutation and histopathological parameters was found. To investigate the clinical relevance of complexes formed by uPAR with its interaction partners, such as uPA or IGF1R in TNBC, the PLA technique was conducted on 174 tumour specimens differentially expressing these biomarkers. The higher the uPAR and IGF1R expression levels were in respective TNBC sample, the higher was the number of uPAR-IGF1R complexes (Fig. 4a), whereas the amount of these complexes was diminished when both interactors were expressed at low levels (Fig. 4b). High amounts of uPAR-IGF1R complexes significantly correlated with histological grade (*p* = 0.0019), Ki67 (*p* = 0.0284), cathepsin B (*p* = 0.0168) or cathepsin D (*p* = 0.0290) (Table 2). IGF1R-uPAR complexes inversely correlated with p27^Kip1^ (*p* = 0.0103), uPA (*p* = 0.0002) and PAI-1 expression (*p* = 0.0001) (Table 2). Complexes of uPAR and uPA significantly correlated with IGF1R (*p* = 0.0032), cathepsin B and D (*p* ≤ 0.0001, respectively) expression and inversely with the tumour size (*p* = 0.0065) (Table 2).

**Table 1:** Significant protein expressions of uPAS components and tumour-associated markers

Tumour marker 1	Tumour marker 2	*p*-values
**uPAR**	IGF1R	0.0011
	Cathepsin B	0.0034
	Cathepsin D	0.0075
	c-Met	0.0229
	IR	0.0215
	Plasminogen	0.0105
**uPA**	PAI-1	≤0.00001
	Cathepsin D	0.0187
	Plasminogen	0.0237
	Ki67	0.0004 (inverse)
**IGF1R**	IR	0.0020
	Cathepsin B	0.0002
	uPARAP	0.0168
	Plasminogen	0.0237
	Ki67	0.0120
	PTEN	≤0.00001
PAI-1	Ki67	0.00006 (inverse)
c-Met	IR	0.0364
	Plasminogen	0.0096
	uPARAP	0.0097
	Ki67	0.0486
IR	Plasminogen	0.0075
	PTEN	0.0083
	Ki67	0.0309

Protein expressions were determined using IHC technique followed by statistical analyses. Correlations of parameters marked in bold are described within the text in more detail

**Table 2:** Significant correlations of uPAR/IGF1R or uPAR/uPA complexes with clinical parameters and tumour-associated markers

PLA complexes	Parameters	*p*-values
uPAR with IGF1R	Histological grade	0.0019
	Ki67	0.0284
	Cathepsin B	0.0168
	Cathepsin D	0.0290
	PAI-1	0.0001 (inverse)
	uPA	0.0002 (inverse)
	p27^Kip1^	0.0103 (inverse)
uPAR with uPA	IGF1R	0.0032
	Cathepsin B	≤0.0001
	Cathepsin D	≤0.0001
	Tumour size	0.0065 (inverse)

## Discussion

The triple-negative breast cancer mostly occurs in young women representing 10–20 % of breast cancers and shows an aggressive phenotype that is correlated with a poor prognosis [2]. To date, these patients are systemically treated with chemo-/radiotherapy and surgery due to the lack of standard therapeutic targets in TNBC, such as ER, PR or HER2 in other breast cancer subtypes. Therefore, novel therapeutics and therapeutic strategies are urgently needed. Although TNBC is a heterogeneous disease [48], it is well known that the major components of the uPA system including uPA, uPAR and PAI-1 are often overexpressed in this BC subtype and may be interesting candidates for targeted therapies [9]. Several tumour-promoting biomarkers such as EGFR, IGF1R, IR and c-Met are also co-overexpressed in TNBC [3, 4, 6–8]. The scope of this study was to demonstrate the potential of a long-term downregulation of uPAR combined with the downregulation of its already known and novel direct interaction partners. Based on preliminary results derived from transient knockdowns of uPAR, uPA and/or IGF1R leading to significantly reduced in vitro proliferation and metastasis, the long-term effects of uPAR- and IGF1R-RNAi were determined in two TNBC model cell lines after stable downregulations of 8 weeks. Since uPAR is associated to the cell membrane only by a GPI anchor, for intracellular signal transduction it has to interact with receptors containing a transmembrane domain. Since IGF1R is associated with uPAR [34, 35] and co-overexpressed in breast cancer [5, 6], here, it was downregulated in combination with uPAR and further analysed to demonstrate a direct interaction of both receptors. In comparison to single RNAi of the receptors, mostly the combined knockdown of uPAR and of IGF1R led to an additively and significantly impaired in vitro proliferation, migration and invasion, in particular of MDA-MB-231 cells. The knockdown of uPA alone or in combination with IGF1R RNAi also affected the malignant potential in vitro; however it was not as prominent as the combination of uPAR and IGF1R. Regarding the knockdown effects on the expression or phosphorylation of several tumour-associated proteins, the effects were rather minor. The phosphorylation of STAT3 or Akt was not changed following long-term knockdowns in both cell lines. Although the in vitro proliferation and metastasis was impaired, it is possible that after 8 weeks of downregulation, the cells adapt to or compensate the lack of uPAR, uPA and/or IGF1R and therefore, the effects on single proteins could not be detected anymore. Nevertheless, in MDA-MB-231 cells, a reduced phosphorylation of c-Met was observed, whereas the cell cycle inhibitor p27^Kip1^ was induced in both cell lines following IGF1R downregulation alone or in combination with uPA RNAi correlating with the results obtained from in vitro proliferation assays. Due to the lack of ER, PR and HER2 but strong uPAR protein expression and exhibiting an invasive phenotype, BT549 cells may be regarded as representative for TNBC. Concerning the protein expressions of IGF1R, MMPs or phosphorylation of c-Met, this cell line differs from MDA-MB-231 cells. In addition, the inhibitory knockdown effects in BT549 cells were not always comparable with those observed in MDA-MB-231 cells. However, our results are in agreement with previous studies demonstrating high molecular heterogeneity in TNBC and in numerous TNBC cell lines [48]. Nevertheless, in BT549 cells, only the in vitro migration was significantly impaired due to RNAi of uPAR or co-RNAi of IGF1R with uPAR or uPA and this was confirmed by immunoblottings showing reduced activation of the metastasis marker MMP2. Neither comparable RNAi studies in BT549 cells nor the combined downregulation of uPAR with IGF1R in TNBC cell lines was demonstrated before. For knockdown studies simultaneously targeting uPAR, uPA or MMPs, mostly the MDA-MB-231, ZR751 or non-TNBC cell lines were applied and the combined knockdown approaches led to significant and additive effects regarding reduced malignancy of tumour cells [31–33] corresponding to our results. Furthermore, although the control shRNAs do not alter the RNA or protein levels of all the target proteins, the in vitro migration and invasion assays of the control approaches show in part unspecific results, which may be associated with the respective cell lines or in vitro assays. Thus, additional in vitro studies are necessary for understanding the tumourigenic signalling cascades in TNBC cells in more detail.

To further investigate the interactions of uPAR with several tumour-promoting proteins, uPA was confirmed and IGF1R was identified as direct interactor in the TNBC cohort and these complexes significantly correlated with cathepsin B and D, whereas IGF1R significantly correlated with cathepsin B and not with cathepsin D. Previous studies have shown cathepsins being correlated with uPAR or uPA and involved in metastasizing processes of breast cancer cells [15, 49] emphasizing the malignant potential of these interactions in TNBC. Furthermore, uPA expression alone significantly correlated with PAI-1, cathepsin D, plasminogen and Ki67 but not with the expression of uPAR, cathepsin B or other clinical parameters, whereas uPA-uPAR complexes significantly correlated with the expression of both cathepsins (B and D), IGF1R and inversely with the tumour size. For the latter correlation we hypothesise a metastasizing effect when the amount of uPAR-uPA complexes is elevated and tumour cells migrate and spread into different organs leading to cell loss within the primary tumour tissue and therefore to a reduced tumour size. IGF1R and uPAR complexes inversely correlated with uPA or PAI-1 expression. This outcome may result from competing impact on the binding site for IGF1R, uPA or PAI-1 with uPAR [50]. The outcomes derived from PLA analyses and statistical correlations with tumour-relevant parameters indicate that not only the overexpression of tumour-promoting markers influences malignancy in TNBC but their interactions with other biomarkers may increase the malignant potential of tumour cells. The idea to impede the interactions of uPAR with uPA or IGF1R, as demonstrated by us, using the combined knockdown of the target proteins and leading to decreased malignancy in vitro may be thinkable for the future, to inhibit tumour progression in vivo by treating TNBC patients with inhibitors targeting these biomarkers in combination. However, further experiments including in vivo animal studies and clinical trials are necessary to endorse our hypothesis.

## Conclusions

The overexpression of tumour-promoting biomarkers impacts malignancy in TNBC but their interactions with other signalling proteins may stronger influence the malignant potential of tumour cells. Impeding the complexes of uPAR with uPA or IGF1R may also strongly inhibit tumour progression in vivo by treating TNBC patients with inhibitors targeting these biomarkers in combination.

## Abbreviations

c-Met, hepatocyte growth factor receptor; ECM, extracellular matrix; EGFR, epidermal growth factor receptor; ER, oestrogen receptor; ERK, extracellular signal-regulated kinase; GPI, glycosylphosphatidyl inositol; HER2, human epidermal growth factor receptor 2; IGF1R, insulin-like growth factor receptor 1; IHC, immunohistochemistry; IR, insulin receptor; MAPK, mitogen-activated protein kinase; MMP, matrixmetalloproteases; PI3K, phosphatidylinositol 3-kinase; PLA, proximity ligation assay; PR, progesterone receptor; RNAi, RNA interference; STAT, signal transducer and activator of transcription; TNBC, triple-negative breast cancer; uPA, urokinase-type plasminogen activator; uPAR, urokinase-type plasminogen activator

## Additional files

Additional file 1: Table S1. Overview of applied primary antibodies for immunohistochemical analyses (IHC) and Western blots (WB). (DOC 53 kb) Additional file 2: Figure S1. Combined RNAi of uPAR and uPA significantly reduced the tumourigenic potential of TNBC cells. **a**: Representative Western blot analysis of uPAR and uPA following RNAi using three different siRNAs per target protein, respectively and of (**b**) MMP2 and 9, (phospho) STAT3, Paxillin. Tubulin was used as loading control. **c**: In vitro viability assays (*n* = 5), **d**: scratch wound assays (*n* = 3) and (**e**) matrigel invasion assays (*n* = 3) are shown 48 h post-transfection. siRNAs for transient downregulations of target proteins or of GAPDH (positive control) and a non-targeting siRNA as negative control were used in triplicates according to previous protocol [37]. The quantifications were determined in relation to mock. Standard deviations and *p*-values are shown. (PDF 329 kb) Additional file 3: Figure S2. Combined RNAi of uPAR and IGF1R significantly reduced the tumourigenic potential of TNBC cells. **a**: Representative Western blot analysis of uPAR and IGF1R following RNAi using three different siRNAs per target protein, respectively and of (**b**) MMP2 and MMP9. Tubulin was used as loading control. **c**: In vitro viability assays (*n* = 5), **d**: scratch wound assays (*n* = 3) and (**e**) matrigel invasion assays (*n* = 3) are shown 48 h post-transfection. siRNAs for transient downregulations of target proteins or of GAPDH (positive control) and a non-targeting siRNA as negative control were used in triplicates according to previous protocol [37]. **f**: Relative mRNA levels of uPAR, IGF1R and uPA following stable RNAi of target proteins determined by qRT-PCR (*n* = 3) are shown. The quantifications were determined in relation to mock control. Standard deviations and p-values are shown. (PDF 336 kb) Additional file 4: Figure S3. Immunohistochemical analysis and PLA of uPAR and IGF1R in MDA-MB-231 and control cells. **a**: Differential protein expressions of uPAR or IGF1R in mock and uPAR-depleted MDA-MB-231 cells or in MCF7 (**b**) and SKBR3 (**d**) cells. **c**: Visualization and quantification of uPAR and IGF1R complexes in MCF7 and in SKBR3 (**e**) cells. (PDF 658 kb)
