# Supplementary material for: uPAR enhances malignant potential of triple-negative breast cancer by directly interacting with uPA and IGF1R
Source: BMC Cancer. 2016 Aug 8;16:615. doi: 10.1186/s12885-016-2663-9 (PMC4977758; doi:10.1186/s12885-016-2663-9)
Supplement: Additional file 1: Table S1. — Overview of applied primary antibodies for immunohistochemical analyses (IHC) and Western blots (WB). (DOC 53 kb) [file 12885_2016_2663_MOESM1_ESM.doc]

**Additional file 1: Table S1**

Overview of applied primary antibodies for immunohistochemical analyses (IHC) and Western blots (WB).

| **Antigen** | **Catalogue number and manufacturer** | **Application** | |
| --- | --- | --- | --- |
|  |  | IHC | WB |
| uPAR | IID7 (41) | 1:500 | 1:500 |
| uPA | 3689, American Diagnostica, Stamford, CT,USA | 1:1000 |  |
| PAI-1 | 3786 & 3785, American Diagnostica, Stamford, CT,USA | 1:500 |  |
| HER2 | A0485, DAKO, Glostrup, DK | 1:300 | 1:5000 |
| PR | A0098, DAKO, Glostrup, DK; | 1:200 |  |
|  | 3157, Cell Signaling, Techn., Beverly, MA, USA |  | 1:1000 |
| ER | Ready to use, Ventana 790-4324 | + |  |
|  | sc-8002, Santa Cruz Biotechn., Heidelberg, DE |  | 1:500 |
| IGF1R | 3027, Cell Signaling, Techn., Beverly, MA, USA | 1:50 | 1:1000 |
| IR | HPA036302, Sigma Aldrich, St. Louis, MO, USA | 1:15 |  |
|  | 3025, Cell Signaling, Techn., Beverly, MA, USA |  | 1:1000 |
| c-Met | ab51067, Abcam Inc, Cambridge, MA, USA | 1:500 | 1:500 |
| phospho-cMet | 3126, Cell Signaling, Techn., Beverly, MA, USA |  | 1:400 |
| Cathepsin B | 3373, Cell Signaling, Techn., Beverly, MA, USA | 1:100 |  |
| Cathepsin D | PDR 004, Diagnostic BioSystems, Ontario, Canada | 1:50 |  |
| Plasminogen | Ab10178, Abcam Inc, Cambridge, MA, USA | 1:35 |  |
| uPARAP | ABIN 951275, antibodies online, Aachen , DE | 1:100 |  |
| Ki67 | Ab15580, Abcam Inc, Cambridge, MA, USA | 1:1000 |  |
| p27^Kip1^ | 610241, BD Biosciences, Lexington, KY, USA | 1:500 |  |
|  | 610242, BD Biosciences, Lexington, KY, USA |  | 1:5000 |
| PTEN | 9559, Cell Signaling, Techn., Beverly, MA, USA | 1:25 | 1:2000 |
| Paxillin | 2542, Cell Signaling, Techn., Beverly, MA, USA |  | 1:500 |
| MMP2 | 4022, Cell Signaling, Techn., Beverly, MA, USA |  | 1:1000 |
| MMP9 | 3852, Cell Signaling, Techn., Beverly, MA, USA |  | 1:1000 |
| STAT3 | 610190, BD Biosciences, Lexington, KY, USA |  | 1:2000 |
| phospho-STAT3 | 9134, Cell Signaling, Techn., Beverly, MA, USA |  | 1:1000 |
| GAPDH | sc-25778, Santa Cruz Biotechn., Heidelberg, DE |  | 1:5000 |
| Tubulin | T5168, Sigma Aldrich, St. Louis, MO, USA |  | 1:5000 |
