# Supplementary figures and images for: uPAR enhances malignant potential of triple-negative breast cancer by directly interacting with uPA and IGF1R
Source: BMC Cancer. 2016 Aug 8;16:615. doi: 10.1186/s12885-016-2663-9 (PMC4977758; doi:10.1186/s12885-016-2663-9)

Additional file 2: Fig. S1

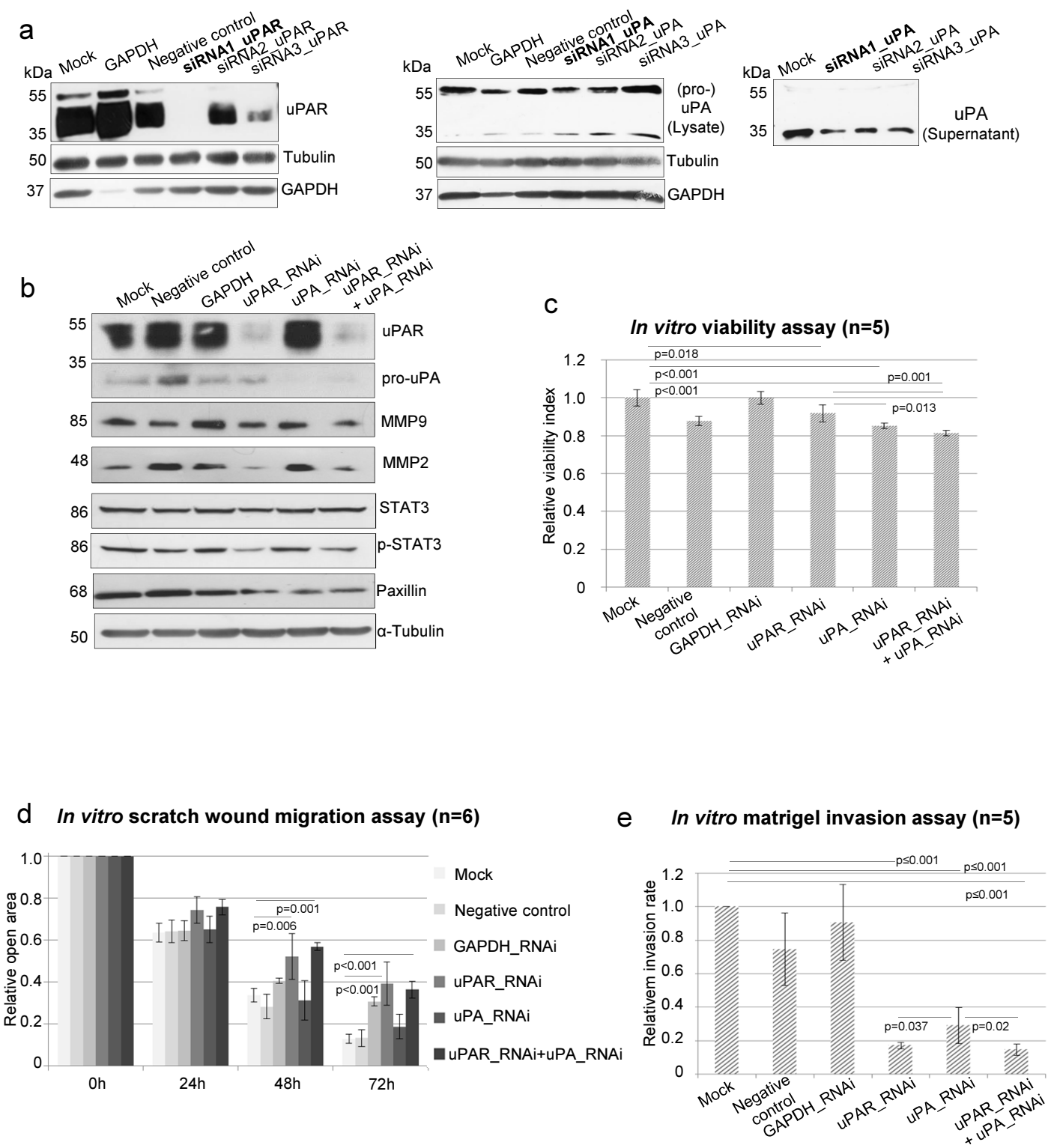

Supplement: Additional file 2: Figure S1. — Combined RNAi of uPAR and uPA significantly reduced the tumourigenic potential of TNBC cells. a: Representative Western blot analysis of uPAR and uPA following RNAi using three different siRNAs per target protein, respectively and of (b) MMP2 and 9, (phospho) STAT3, Paxillin. Tubulin was used as loading control. c: In vitro viability assays (n = 5), d: scratch wound assays (n = 3) and (e) matrigel invasion assays (n = 3) are shown 48 h post-transfection. siRNAs for transient downregulations of target proteins or of GAPDH (positive control) and a non-targeting siRNA as negative control were used in triplicates according to previous protocol [37]. The quantifications were determined in relation to mock. Standard deviations and p-values are shown. (PDF 329 kb) [file 12885_2016_2663_MOESM2_ESM.pdf]

# Additional file 3: Fig. S2

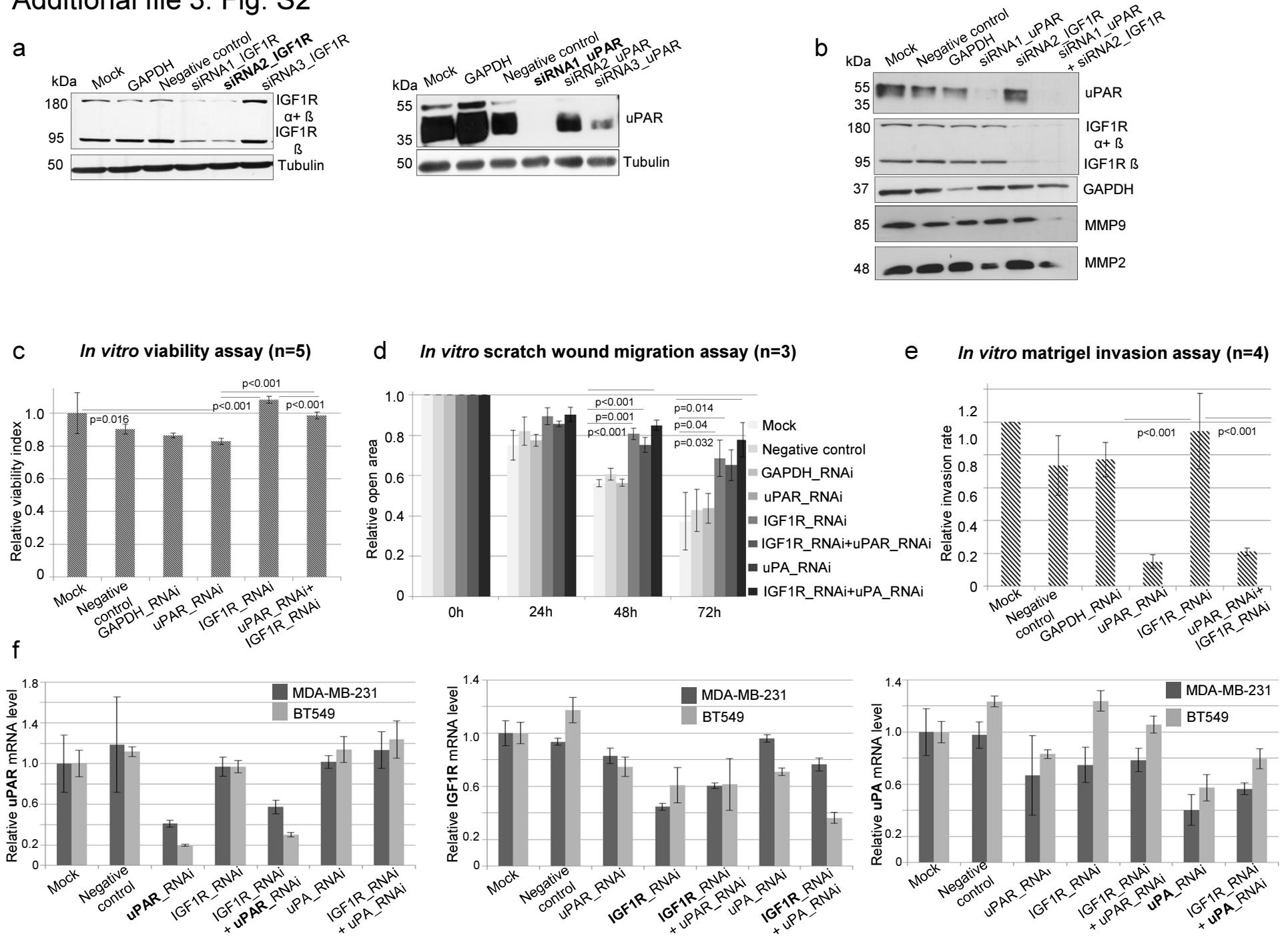

Supplement: Additional file 3: Figure S2. — Combined RNAi of uPAR and IGF1R significantly reduced the tumourigenic potential of TNBC cells. a: Representative Western blot analysis of uPAR and IGF1R following RNAi using three different siRNAs per target protein, respectively and of (b) MMP2 and MMP9. Tubulin was used as loading control. c: In vitro viability assays (n = 5), d: scratch wound assays (n = 3) and (e) matrigel invasion assays (n = 3) are shown 48 h post-transfection. siRNAs for transient downregulations of target proteins or of GAPDH (positive control) and a non-targeting siRNA as negative control were used in triplicates according to previous protocol [37]. f: Relative mRNA levels of uPAR, IGF1R and uPA following stable RNAi of target proteins determined by qRT-PCR (n = 3) are shown. The quantifications were determined in relation to mock control. Standard deviations and p-values are shown. (PDF 336 kb) [file 12885_2016_2663_MOESM3_ESM.pdf]

Additional file 4: Fig. S3

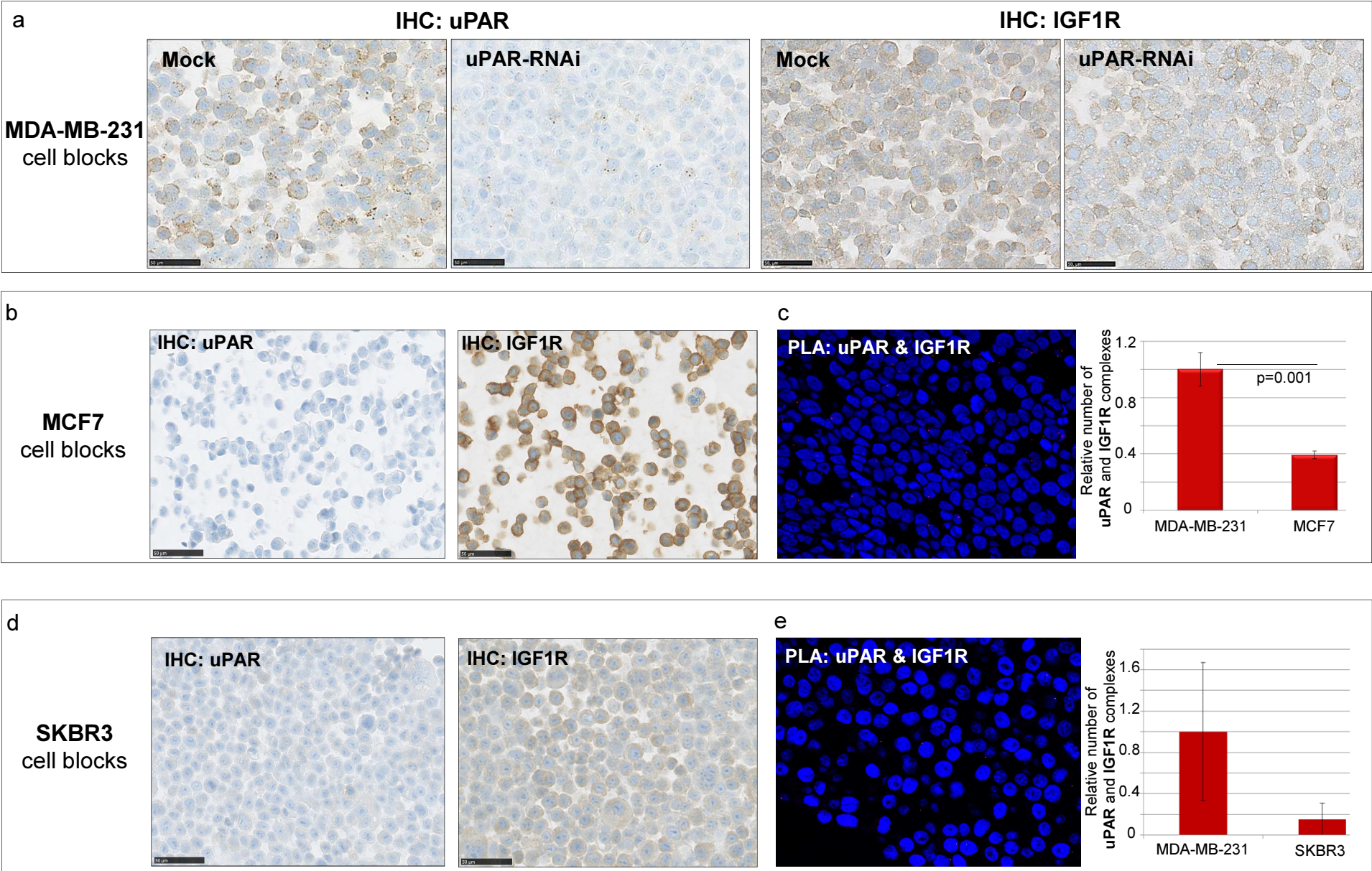

Supplement: Additional file 4: Figure S3. — Immunohistochemical analysis and PLA of uPAR and IGF1R in MDA-MB-231 and control cells. a: Differential protein expressions of uPAR or IGF1R in mock and uPAR-depleted MDA-MB-231 cells or in MCF7 (b) and SKBR3 (d) cells. c: Visualization and quantification of uPAR and IGF1R complexes in MCF7 and in SKBR3 (e) cells. (PDF 658 kb) [file 12885_2016_2663_MOESM4_ESM.pdf]
